# Patient and public involvement and engagement in target trial emulation framework: a scoping review protocol

**DOI:** 10.1136/bmjopen-2025-113432

**Published:** 2026-03-24

**Authors:** Isaac J Egesa, Faye D Baldwin, Molly Wells, Michelle Maden, Gashirai K Mbizvo, Anthony Guy Marson, Catrin Tudur-Smith

**Affiliations:** 1 University of Liverpool Department of Health Data Science, Liverpool, England, UK; 2 Faculty of Biology, Medicine and Health, The University of Manchester Division of Informatics, Imaging and Data Sciences, Manchester, England, UK; 3 Liverpool Interdisciplinary Neuroscience Centre, University of Liverpool, Liverpool, England, UK; 4 Liverpool Centre for Cardiovascular Science, University of Liverpool, Liverpool, UK; 5 The Walton Centre NHS Foundation Trust, Liverpool, England, UK

**Keywords:** Observational Study, Review, STATISTICS & RESEARCH METHODS, EPIDEMIOLOGY

## Abstract

**Abstract:**

**Introduction:**

Target trial emulation (TTE) has emerged as a methodological framework to strengthen causal inference from observational health data when randomised controlled trials are infeasible. The credibility of TTE studies depends not only on rigorous design and transparent reporting, but also on their relevance and acceptability to patients and the public. Patient and public involvement and engagement (PPIE) has been shown to enhance the relevance, transparency and impact of health research by shaping research priorities, informing study design and ensuring outcomes reflect patient perspectives. However, the extent to which PPIE has been incorporated into TTE studies remains unclear. This scoping review aims to systematically map the use and reporting of PPIE in published TTE studies.

**Methods and analysis:**

This review will follow the Joanna Briggs Institute methodology for scoping reviews and will be reported according to the Preferred Reporting Items for Systematic Review and Meta-Analysis extension for Scoping Reviews checklist. We will search MEDLINE (Ovid) and Embase (Ovid) from January 2011 to present, limited to English-language publications. Eligible studies will be studies that self-identify as using the TTE framework and report empirical analyses of health outcomes using observational or trial data. We will exclude protocols, methodological or simulation-only studies, preprints, conference abstracts and grey literature. Three reviewers will independently screen titles and abstracts, and then full texts, with disagreements resolved by discussion or adjudication. Data extraction will include study characteristics and PPIE information guided by the Guidance for Reporting Involvement of Patients and the Public 2-Short Form checklist. Findings will be summarised using descriptive statistics, tables, figures and narrative synthesis.

**Ethics and dissemination:**

Ethics approval is not required, as this review will use publicly available data. Results will be disseminated through a peer-reviewed publication and presented at conferences.

**STRENGTHS AND LIMITATIONS**

- Guidance for Reporting Involvement of Patients and the Public 2-Short Form checklist will be used to assess the quality and completeness of patient and public involvement and engagement reporting.
- Joanna Briggs Institute methodology for scoping reviews with transparent, reproducible search and screening procedures will be used.
- At least two independent reviewers will conduct screening and data extraction to minimise selection bias.
- Inclusion will be restricted to studies that self-identify as using the target trial emulation framework, potentially omitting studies without explicit labelling.
- The search is limited to studies published in English, which may exclude relevant non-English publications.

## Introduction

 Decision-making in healthcare requires timely and relevant evidence. Randomised controlled trials (RCTs) remain the gold standard for inference, but they are not always feasible due to cost, time, generalisability or ethical constraints. The analysis of routinely collected health data sources, such as electronic health records, disease registries or administrative datasets, offers an alternative route to generating evidence. While these sources are invaluable, they are susceptible to systematic biases, including confounding, selection bias and immortal-time bias, which can undermine causal interpretation if left unaddressed.^1^,^6^

The target trial emulation (TTE) framework provides a structured approach to strengthen causal inference from observational data.^7^ When applied correctly, the results from a TTE can mirror those of an RCT, as exemplified by the results of a TTE on dialysis in chronic kidney disease being similar to those of the randomisedInitiating Dialysis Early and Late (IDEAL) trial.^8 9^ TTE was first popularised by Hernán and Robins.^10^ The TTE framework requires explicit specification of the protocol of a hypothetical randomised trial (the ‘target trial’), which may or may not be based on an existing RCT, and then mapping that protocol to the available data. By requiring a priori definitions of eligibility criteria, treatment strategies, assignment procedures, follow-up period, study outcome, causal contrast or estimand and analysis plan, TTE clarifies assumptions and ameliorates pitfalls in inference from observational data. Applications of TTE have expanded across comparative effectiveness research, pharmacoepidemiology and policy evaluation, illustrating its pragmatic value when randomised evidence is unavailable.^11^,^18^ For example, a review of 200 TTE studies published between 2012 and 2022 found that more than three-quarters of the studies appeared more recently, between 2020 and 2022.^12^ Reviewers have also reported substantial gaps in TTE, including failing to describe how the target trial is emulated, to present the protocol clearly and to address confounders.^11 12^ However, the credibility of TTE may not only depend on methodological rigour and transparent reporting, but also on the relevance and acceptability of its design choices and generalisability of assumptions to patients and the public.^19^

In parallel with TTE’s steadfast growth, there has been growing recognition of patient and public involvement and engagement (PPIE) in high-quality health research. PPIE refers to an active partnership with patients, carers and members of the public across the research process.^20^ For example, PPIE representatives can be involved in activities such as helping to define research questions, developing protocols, identifying study outcomes, reviewing dissemination materials or preparing lay summaries. PPIE advocates argue that it can enhance the relevance, acceptability and legitimacy of health research outputs.^21 22^ To promote better and complete reporting of PPIE, tools such as the Guidance for Reporting Involvement of Patients and Public (GRIPP2) checklists have been developed.^23^ Journals and funders are also increasingly encouraging or requiring PPIE statements.^24 25^ Nevertheless, previous research consistently reports that PPIE is frequently under-reported or described with insufficient detail to appraise its purpose, context and impact on design, analysis, interpretation or dissemination in health research.^26 27^

Despite the advanced methodology and growth of TTE (including reporting guidelines)^19^ and heightened PPIE awareness, to our knowledge, there has not been a systematic mapping of how PPIE is reported, described and operationalised in TTE studies. While we recognise that PPIE may occur without being reported,^23 28^ without such a review, the extent to which PPIE is incorporated into TTE research and how this might be improved remains unknown. This represents a consequential gap as key TTE design decisions, for example, selecting meaningful outcomes, defining the causal question and estimand, specifying time zero, treatment strategies and judging the acceptability of analytical trade-offs, are value-laden and likely to benefit from the perspectives of people with lived experience or members of the public.^19 29^ A lack of meaningful PPIE in TTE studies risks misalignment with priorities important to patients and the public, potentially leading to evidence from this design being irrelevant, unacceptable and poorly taken up downstream.

To provide a comprehensive picture of PPIE in TTE, we aim to conduct a scoping review to address the following questions:

- What proportion of published TTE studies report any PPIE? How does reporting vary by study characteristics and calendar time?
- In which stages of the TTE lifecycle (eg, causal question/estimand definition, protocol specification, protocol mapping to observational data, bias/confounder control method selection, statistical analysis, reporting and interpretation and dissemination stage) has PPIE been involved?
- What roles (eg, advisory, co-production, co-analysis, dissemination) and rationales for PPIE are described or operationalised in TTE studies, and how completely are they reported (eg, per GRIPP2-Short Form (SF) domains)?

## Methods

The review will be guided by the Joanna Briggs Institute (JBI) methodology for developing scoping review protocols, literature search, data extraction and synthesis.^30^ It will then be reported following the Preferred Reporting Items for Systematic Review and Meta-Analysis extension for Scoping Reviews (PRISMA-ScR) checklist.^31^ The protocol is registered in the Open Science Framework (OSF) (osf.io/5tx2q). Any protocol deviations with the dates and rationale will be documented in the OSF record and reported in the final review. The review is planned to be conducted from November 2025 to March 2026.

### Eligibility criteria

The inclusion criteria will be structured using the JBI framework, which includes participants, concepts and context.

#### Participants

Eligible studies will include published empirical research involving patients, carers, members of the public or individuals with lived experience of any health condition who engage in the design, conduct or dissemination of TTE studies. TTE studies without PPIE will also be included, as one of our objectives is to quantify the proportion of TTE studies that report PPIE.

#### Concept

The concept is the PPIE in studies explicitly identifying themselves as TTE. Eligible studies must (1) self-describe as TTE, target trial or use the target trial framework and (2) report empirical analyses of health-related outcomes using observational or randomised trial data. We will exclude methodological, theoretical or simulation studies and TTE protocol papers, as these do not represent completed empirical analyses of health outcomes.

#### Context

We will not restrict our inclusion criteria to any health condition, healthcare setting or population group studied. The review will include studies from all countries but will be limited to English-language publications.

#### Type of studies

We will include only peer-reviewed journal articles. Grey literature, conference abstracts, editorials, commentaries, theses and preprints will be excluded.

### Search strategy

The search strategy will be developed in consultation with an experienced information specialist. We will search MEDLINE (Ovid) and Embase (Ovid) from January 2011 to present, restricting the search to English-language publications. The start date of 2011 was selected because the TTE concept was introduced in the early 2000s,^10^ gained traction after 2010 and reporting standards for PPIE (eg, GRIPP) were developed around the same time.^32^ The preliminary search strategy will combine controlled vocabulary and free-text terms, including: ‘target trial emulation’, ‘target trial’, ‘target trial framework’, ‘target trial emulat*’ and close variants adopted from previous reviews.^12 14 15 18^ This search strategy will be refined based on piloting against a set of ~10 known TTE studies to ensure high sensitivity. The full draft search strategy is provided in the online supplemental file 1. In addition to database searching, we will conduct backward citation of included studies and relevant reviews to identify additional eligible studies.

### Study selection

Search results will be imported into EndNote reference manager^33^ for deduplication and then uploaded into Rayyan^34^ for screening. Screening will proceed in two stages: (1) titles and abstract screening, followed by (2) full-text screening. Three reviewers (IJE, FDB, MW) will independently screen the articles against eligibility criteria. One reviewer (IJE) will screen all the articles, while the other two reviewers will each screen a randomly assigned 50% subset generated in R using the sample() function.^35^ This approach will ensure independent verification of the screening process while reducing workload in the event of a large number of included studies. Disagreements will be resolved through discussion or, if necessary, adjudication by a senior reviewer. Reasons for exclusion at full-text stage will be documented. The study selection process, details of the search results and reasons for exclusion at the full-text stage will be presented in the PRISMA flow diagram according to the PRISMA-ScR checklist.

### Data extraction

We will use a piloted Microsoft Excel template to record extracted data. The planned extraction data include:

- Study characteristics: first author, DOI (Digital Object Identifier), journal, country, year of publication, clinical domain, data source, funding source.
- PPIE variables: PPIE reporting (yes/no), aim of PPIE, section reported, stage of TTE lifecycle, who was involved, recruitment method, role, method of involvement, changes due to PPIE, challenges/benefits, documented impact, compensation, tokenism statements and GRIPP2-SF completeness.

One reviewer (IJE) will extract data from all the included studies, while the other two reviewers (FDB and MW) will each independently extract data from 50% of the studies, selected at random. Discrepancies will be reconciled by consensus. We will adjust the extraction details during the extraction process if needed and report the changes in the OSF and final manuscript.

### Defining and Identifying PPIE in included studies

We will define and classify PPIE using the GRIPP2-SF checklist. The GRIPP2-SF includes five items on aims, methods, outcomes, discussion and critical perspective and is suitable for studies where patient and public involvement is a secondary focus.^23^ During extraction, we will search for PPIE evidence, including the author/contributor list, acknowledgements, methods, patient/public involvement section, funding statements, ethics, discussion, author affiliations and dissemination statements. Potential indicators of PPIE include mention of patient/public advisors, expert by experience, lay representatives/member, patient or carer representatives/member, patient research consultant, public representative/member and user/service user.^36^

### Data synthesis and analyses

We will summarise findings using descriptive statistics (counts, proportions, trends over time) and present them in tables and figures. Narrative synthesis will be used to describe the variations of PPIE reporting across TTE studies. Analyses will be conducted in R statistical software.^35^

### Patient and public involvement

Patients or members of the public were not involved in the design, conduct or reporting of this protocol. However, public contributors will be involved throughout the scoping review process, as described below.

We will recruit a minimum of two contributors with the option to include more depending on availability and interest. Having at least two contributors will ensure diversity of perspectives and help avoid overreliance on a single voice, while remaining feasible within the project’s scope and resources. Contributors will be recruited through established networks (eg, Trial Methodology Research Partnership (TMRP)—Network Hubs: TMRP Working Groups), ensuring they have experience of methodology-related PPIE to minimise training needs. Recruitment will be conducted transparently (via advertisement or targeted invitation) and details of engagement, expected hours and timelines will be shared in advance. Recruitment will start following protocol registration in the OSF, with the aim of engaging contributors before the screening phase begins.

Contributors will engage in the following ways:

- Review and comment on the scoping review protocol, data extraction, to provide feedback on the clarity, relevance and usefulness of the planned review, including ensuring information collected captures aspects of PPIE in TTE that are relevant to patients and the public. The feedback will be discussed with the review team and incorporated into revisions. Any changes resulting from PPIE feedback will be recorded as amendments in the OSF and reported in the final publication.
- Participate in at least one virtual meeting to discuss the scoping review and refine data extraction template if necessary.
- Contribute to the synthesis and interpretation of the draft findings and lay summaries to support interpretations, challenge overly technical framing and identify implications for wider audiences.
- Where possible, assist in developing lay dissemination to broader non-specialist audiences.

Public contributors will be reimbursed for their time in line with the National Institute for Health Research recommendations (£25/hour and £5 for internet) for the hours committed in the review process. No additional expenses are expected from this engagement, since all activities will be conducted online. Contributors who meet the International Committee of Medical Journal Editors criteria for authorship will be offered coauthorship on final review publication. All contributors will be appropriately acknowledged, regardless of authorship status. All protocol amendments, PPIE contributions and their impact on the review process will be reported in the final publication using the GRIPP2-SF checklist.

### Quality assessment

The scoping review does not assess the study’s risk of bias as stipulated by the JBI guidelines; therefore, we will not conduct a formal risk of bias or methodological quality assessment. However, the completeness and quality of PPIE reporting in included studies will be assessed using the GRIPP2-SF checklist.

### Ethics and dissemination

The scoping reviews do not require ethics approval because the data used is publicly available. The review findings will be presented at conferences and seminars, and in a peer-reviewed open-access journal article.

## Supplementary material

10.1136/bmjopen-2025-113432 online supplemental file 1
