# Supplementary material for: Patient and public involvement and engagement in target trial emulation framework: a scoping review protocol
Source: BMJ Open. 2026 Mar 24;16(3):e113432. doi: 10.1136/bmjopen-2025-113432 (PMC13034334; doi:10.1136/bmjopen-2025-113432)
Supplement: online supplemental file 1 [file bmjopen-16-3-s001.docx]

**Supplementary file**

**Search strategy**

Ovid MEDLINE

1. (emulat* or "emulated trial*" or "emulated randomi*" or "trial emulat* or pragmatic emulate*").mp.
2. ("target trial*" or "target clinical trial*" or "target-trial*").mp.
3. (pseudotrial* or pseudo-trial* or "pseudo trial*" or hypothetical trial* or hypothetical-trial).mp.
4. ((mimic* or simulate or replicat*) adj3 trial*).mp.
5. or/1-4
6. exp Observational Study/ or exp Cohort Studies/
7. (observational adj2 (stud*or research or data)).mp.
8. (real world or real-world or rwd).mp.
9. (routinely-collected or routinely collected).mp.
10. (electronic health record* or electronic medical record* or EHR or EMR).mp.
11. (claims data or claims-data or administrative claims or administrative data).mp.
12. (registr*or patient registr* or patient-regist*).mp.
13. (observational adj (study or studies or design or analysis or analyses)).ti,ab,kf.
14. (cohort* adj (study or studies or design or analysis or analyses)).ti,ab,kf.
15. Longitudinal Studies/ or Prospective Studies/ or Follow-Up Studies/
16. ((follow up or follow-up) adj (study or studies or design or analysis or analyses)).ti,ab,kf.
17. (Observational Study or Validation Study or Clinical Study or Evaluation Study or Comparative Study).pt.
18. (prospective* adj (study or studies or design or analy*)).ti,ab,kf.
19. ((longitudinal or long-term or (long adj term)) adj (study or studies or design or analysis or analyses or data)).ti,ab,kf.
20. (population adj (study or studies or analysis or analyses)).ti,ab,kf.
21. or/6-20
22. 5 and 21
23. limit 22 to yr="2011 -Current"

**EMBASE**

1. (emulat* or "emulated trial*" or "emulated randomi*" or "trial emulat*" or pragmatic emulate*).mp.
2. ("target trial*" or "target clinical trial*" or "target-trial*").mp.
3. (pseudotrial* or pseudo-trial* or "pseudo trial*" or hypothetical trial* or hypothetical-trial).mp.
4. ((mimic* or simulate or replicat*) adj3 trial*).mp.
5. or/1-4
6. exp observational study/ or exp cohort analysis/
7. (observational adj2 (stud* or research or data)).mp.
8. (real world or real-world or rwd).mp.
9. (routinely-collected or routinely collected).mp.
10. (electronic health record* or electronic medical record* or EHR or EMR).mp.
11. (claims data or claims-data or administrative claims or administrative data).mp.
12. (registr* or patient registr* or patient-regist*).mp.
13. (observational adj (study or studies or design or analysis or analyses)).ti,ab,kw.
14. (cohort* adj (study or studies or design or analysis or analyses)).ti,ab,kw.
15. exp longitudinal study/ or exp prospective study/ or exp follow up/
16. ((follow up or follow-up) adj (study or studies or design or analysis or analyses)).ti,ab,kw.
17. (Observational Study or Validation Study or Clinical Study or Evaluation Study or Comparative Study).pt.
18. (prospective* adj (study or studies or design or analy*)).ti,ab,kw.
19. ((longitudinal or long-term or (long adj term)) adj (study or studies or design or analysis or analyses or data)).ti,ab,kw.
20. (population adj (study or studies or analysis or analyses)).ti,ab,kw.
21. or/6-20
22. 5 and 21
23. limit 22 to yr="2011 -Current"
