# Supplementary material for: Patient and public involvement and engagement in target trial emulation framework: a scoping review protocol
Source: BMJ Open. 2026 Mar 24;16(3):e113432. doi: 10.1136/bmjopen-2025-113432 (PMC13034334; doi:10.1136/bmjopen-2025-113432)
Supplement: Supplementary file 2 [file bmjopen-2025-113432.reviewer_comments.pdf]

PEER REVIEW HISTORY

BMJ Open publishes all reviews undertaken for accepted manuscripts. Reviewers are asked to complete a checklist review form and are provided with free text boxes to elaborate on their assessment. These free text comments are reproduced below.

ARTICLE DETAILS

Title (Provisional)

Patient and public involvement and engagement in target trial emulation framework: a scoping review protocol

Authors

Egesa, Isaac; Baldwin, Faye D; Wells, Molly; Maden, Michelle; Mbizvo, Gashirai K; Marson, Anthony Guy; Tudur-Smith, Catrin

---

VERSION 1 - REVIEW

Reviewer 1

Name Davies, Gwyneth

Affiliation University College London Great Ormond Street Institute of Child Health, Population Policy and Practice Research and Teaching Department

Date 23-Dec-2025

COI I note funding by MRC TMRP DTP. I am a member of MRC TMRP health informatics working group. I confirm that I have had no involvement in this manuscript at any stage, nor collaborate with any of the authors.

Thank you for inviting me to review this protocol paper by Egesa et al, entitled 'Patient and public involvement and engagement in target trial emulation framework: a scoping review protocol'. It is a well written and succinct manuscript, on a topic of relevance to the field. The introduction briefly summarises the TTE framework and PPIE in research. Methods are in general clearly defined, with inclusion of search strategy as supplementary file.

Major comment:

PPIE involvement section, page 8: I note the statement "Public contributors will be involved throughout the scoping review process", and that contributors will "Review and comment on the scoping review protocol".

Given the topic, it is disappointing to see that there are not yet any PPIE members identified (nor included as an author). Please justify why proceeding with the protocol publication now is the optimal approach, rather than after taking any such PPIE input into account.

Minor comments:

Page 3 Strengths and limitations: For clarity, please separate into strengths and limitations, or make clearer when you are referring to a limitation (e.g. for point 3, "Includes only studies that self-identify as using the target trial framework").

Page 3, line 55 states "The TTE framework requires explicit specification of the protocol of a hypothetical randomised trial (the “target trial”) and then mapping that protocol to the available data". However, the target trial does not need to be a hypothetical trial, it can be an actual RCT. The authors in fact point this out in an earlier sentence referring to the IDEAL trial. Suggest minor rewording of sentence for clarification purposes.

'PPIE' is referred to as if it is an object rather than a person or persons. For example, sentence on page 4 line 31-33 "For example, PPIE can be involved in activities such as helping to define research questions, developing protocols, identifying study outcomes, reviewing dissemination materials or preparing lay summaries." Recommend adding e.g. 'representatives' or similar after 'PPIE', or replacing 'can be involved' with 'can involve'.

Page 4 line 55. The interpretation of the sentence "This is a consequential gap in the use of TTE in observational studies" with respect to the prior sentence about mapping PPIE activities, is currently unclear. Please review /clarify.

Page 6. How will TTE protocol papers be handled? I presume they would be excluded given no health-related outcomes, but given increase in protocol publications for TTEs, it would be good to clarify.

Page 7 Data extraction section: "We will use a piloted Microsoft Excel to record extracted data". Please review this sentence, as it appears to be missing a word such as 'template' or 'tool'.

References: Please review list to ensure citations are complete. For example, the JAMA target statement (reference 19) only has journal and year.

**Reviewer**	**2**
**Name**	**Zippel-Schultz, Bettina**
**Affiliation**	**The German Foundation for the Chronically Ill**
**Date**	**20-Jan-2026**
**COI**	**None**

This review protocol focuses on the intersection between research, particularly TTE, and the involvement of patients and the public in research (PPIE). This is a timely and relevant topic. Overall, the protocol is methodologically sound and ready for publication. The manuscript successfully demonstrates that significant decisions regarding TTE design are value-laden

and would benefit from the insights of patients and the public, thereby strengthening the conceptual contribution. The background section clearly explains the methodological significance of TTE and the normative and practical role of PPIE. The research questions are well-defined, coherent, and appropriate for a scoping review. The planned involvement of public contributors throughout the review process (protocol refinement, interpretation and dissemination) demonstrates strong alignment between the topic and its practical application, thereby enhancing credibility.

Minor clarifications and refinements could possibly be made.

- Limiting the study to research that explicitly identifies itself as TTE may result in relevant work that adheres to TTE principles but does not use this designation being overlooked. While the authors acknowledge this implicitly, the protocol would be strengthened by a more explicit discussion of the potential implications for completeness and generalisability.

- The protocol focuses on reported PPIE, which is appropriate, but equates the absence of reports with the absence of participation. This is a well-known issue in PPIE research and should be mentioned briefly.

VERSION 1 - AUTHOR RESPONSE

REVIEWER 1

Major comment:
PPIE involvement section, page 8: I note the statement "Public contributors will be involved throughout the scoping review process", and that contributors will "Review and comment on the scoping review protocol".
Given the topic, it is disappointing to see that there are not yet any PPIE members identified (nor included as an author). Please justify why proceeding with the protocol publication now is the optimal approach, rather than after taking any such PPIE input into account.

Response:

We thank the reviewer for this thoughtful and important observation, which reflects the principles that motivated our review protocol. We appreciate the opportunity to justify the timing of the protocol publication relative to PPIE engagement. While we agree that engaging contributors from the outset would be ideal, we chose to proceed with publication at this stage for the following reasons:

First, our approach follows an established model for scoping and systematic review methodology, in which a protocol is developed, registered, and/or published to provide a transparent, citable baseline of planned methods. This reference document (protocol) then allows any subsequent changes, including those arising from PPIE input, to be explicitly documented, tracked and reported in the final review paper. We believe this approach will enhance the transparency and impact of PPIE contributions.

Second, by registering the protocol on the Open Science Framework (OSF) prior to PPIE engagement, we establish a documented starting point and thus, PPIE contributors may not be involved at this stage. We have revised to make this clearer by stating: "*Recruitment will start following protocol registration in the OSF, with the aim of engaging contributors before the screening phase begins*") [PPIE involvement section].

Third, our protocol describes an iterative PPIE engagement plan. As the reviewer acknowledges, contributors will be "involved throughout the scoping review process," with reviewing and commenting on the protocol as one of their first activities. We acknowledge that apart from stating that "*Any protocol deviations with the dates and rationale will be documented in the OSF record and reported in the final review's manuscript*" [page 5], we did not explicitly state that feedback from this activity would inform revisions to the registered protocol before screening begins. We have now revised the manuscript to clarify this point to read: "*Any changes resulting from PPIE feedback will be recorded as amendments in the OSF and reported in the final publication"*.

Fourth, recruitment of PPIE contributors through established networks such as TMRP requires sharing clear documentation of the project scope, time commitment, and expectations. This information can only be communicated effectively through a well-developed protocol.

Fifth, regarding authorship considerations, we are committed to appropriately recognising PPIE contributors, including offering co-authorship on the final review paper where their contributions meet ICMJE criteria. At this protocol stage, contributors have not yet engaged in activities warranting authorship. We have now revised the manuscript to read "*Contributors who meet the International Committee of Medical Journal Editors (ICMJE) criteria for authorship will be offered co-authorship on final review publication. All contributors will be appropriately acknowledged, regardless of the authorship status*"

Minor comments:
Reviewer 1 comment
Page 3 Strengths and limitations: For clarity, please separate into strengths and limitations, or make clearer when you are referring to a limitation (e.g. for point 3, "Includes only studies that self-identify as using the target trial framework").

Response:

We thank the reviewer for this constructive suggestion. We do agree that the original presentation lacked clarity on which points are strengths versus limitations. We have now clearly separated this with sub-headings and rephrased the limitations to highlight potential sources.

Reviewer 1 comment
Page 3, line 55 states "The TTE framework requires explicit specification of the protocol of a hypothetical randomised trial (the "target trial") and then mapping that protocol to the available data". However, the target trial does not need to be a hypothetical trial, it can be an actual RCT. The authors in fact point this out in an earlier sentence referring to the IDEAL trial. Suggest minor rewording of sentence for clarification purposes.

Response:

We thank the reviewer for this astute observation. The reviewer is correct that the target trial in TTE does not need to be hypothetical only, but can also be based on an actual RCT, as the case of the IDEAL trial cited. We acknowledge the original wording was imprecise and, we have revised the sentence to read: *The TTE framework requires explicit specification of the protocol a hypothetical randomised trial (the "target trial"), which may or may not be based on an existing RCT, and then mapping that protocol to the available data.*

Reviewer 1 comment
'PPIE' is referred to as if it is an object rather than a person or persons. For example, sentence on page 4 line 31-33 "For example, PPIE can be involved in activities such as helping to define research questions, developing protocols, identifying study outcomes, reviewing dissemination materials or

preparing lay summaries." Recommend adding e.g. 'representatives' or similar after 'PPIE', or replacing 'can be involved' with 'can involve'.
Response:

We thank the reviewer for this observation. The reviewer is correct that we used PPIE as an activity or process rather than individuals. We have revised the sentence to read: "*PPIE representatives can be involved in activities such as helping to define research questions, developing protocols, identifying study outcomes, reviewing dissemination materials or preparing lay summaries*". We have also reviewed the manuscript for similar instances and ensured consistent use of person-centred terminologies.

Reviewer 1 comment
Page 4 line 55. The interpretation of the sentence "This is a consequential gap in the use of TTE in observational studies" with respect to the prior sentence about mapping PPIE activities, is currently unclear. Please review /clarify.

Response:
We are grateful to the reviewer for pointing out this unclear sentence. We agree that the connection between the statement and the prior sentence on mapping was unclear. Our intended meaning was that the absence of a systematic review (scoping in our case) means we currently cannot understand whether and how PPIE is being incorporated into TTE research, and this matters because TTE involves decisions that would benefit from public contributors.

We have revised the text to clarify the logical flow, and now it reads:

"Despite the advanced methodology and growth of TTE (including reporting guidelines)[19] and heightened PPIE awareness, to our knowledge, there has not been a systematic mapping of how PPIE is reported, described, and operationalised in TTE studies. Without such a review, the extent to which PPIE is incorporated into TTE research and how this might be improved remains unknown. This represents a consequential gap as key TTE design decisions, for example, selecting meaningful outcomes, defining the causal question and estimand, specifying time zero, treatment strategies, and judging the acceptability of analytic trade-offs, are value-laden and likely to benefit from the perspectives of people with lived experience or members of the public."

Reviewer 1 comment
Page 6. How will TTE protocol papers be handled? I presume they would be excluded given no health-related outcomes, but given increase in protocol publications for TTEs, it would be good to clarify.

Response:
We thank the reviewer for this comment. Indeed, the protocols would be excluded because of not reporting health-related outcomes. We acknowledge that, this was not stated and appreciate an opportunity to clarify. We have now revised the concept section page 6 to address this.

Reviewer 1 comment
Page 7 Data extraction section: "We will use a piloted Microsoft Excel to record extracted data". Please review this sentence, as it appears to be missing a word such as 'template' or 'tool'.
Response:
We thank the reviewer for identifying this error. We have revised the sentence to read: "*We will use a piloted Microsoft Excel template to record extracted data*"

Reviewer 1 comment
References: Please review list to ensure citations are complete. For example, the JAMA target statement (reference 19) only has journal and year.

Response:

We thank the reviewer for identifying this oversight. We have reviewed the reference list and corrected the incomplete citation for reference 19 to include all required details. We have also reviewed all other references to ensure completeness and accuracy throughout the manuscript.

REVIEWER 2

Comments to the Author:
This review protocol focuses on the intersection between research, particularly TTE, and the involvement of patients and the public in research (PPIE). This is a timely and relevant topic. Overall, the protocol is methodologically sound and ready for publication. The manuscript successfully demonstrates that significant decisions regarding TTE design are value-laden and would benefit from the insights of patients and the public, thereby strengthening the conceptual contribution. The background section clearly explains the methodological significance of TTE and the normative and practical role of PPIE. The research questions are well-defined, coherent, and appropriate for a scoping review. The planned involvement of public contributors throughout the review process (protocol refinement, interpretation and dissemination) demonstrates strong alignment between the topic and its practical application, thereby enhancing credibility.

Minor comments

Reviewer 2 comment
- Limiting the study to research that explicitly identifies itself as TTE may result in relevant work that adheres to TTE principles but does not use this designation being overlooked. While the authors acknowledge this implicitly, the protocol would be strengthened by a more explicit discussion of the potential implications for completeness and generalisability.
Response:

We thank the reviewer for this thoughtful observation. We agree that limiting inclusion to studies that self-identify as TTE may exclude research that applies TTE principles without using this specific terminology. This approach was chosen to ensure reproducibility and feasibility, consistent with previous TTE reviews. However, we recognise this has implications for completeness and generalisability, which we will discuss explicitly in the discussion section of the final review paper.

Reviewer 2 comment
The protocol focuses on reported PPIE, which is appropriate, but equates the absence of reports with the absence of participation. This is a well-known issue in PPIE research and should be mentioned briefly.
Response:

We thank the reviewer for this important methodological insight. Published literature shows that the absence of PPIE in reports does not equate to the absence of PPIE activities. We have added this acknowledgement in the introduction paragraph 4, and cited the appropriate reference to support this.
"*While we recognise that PPIE may occur without being reported…".*
